# Photoactive Silver Nanoagents for Backgroundless Monitoring and Precision Killing of Multidrug-Resistant Bacteria

**DOI:** 10.7150/ntno.62364

**Published:** 2021-06-01

**Authors:** Zhiwen Xu, Cai Zhang, Yunjian Yu, Wenshuai Li, Zhuang Ma, Jingjing Wang, Xinge Zhang, Hongmei Gao, Dingbin Liu

**Affiliations:** 1 State Key Laboratory of Medicinal Chemical Biology, Research Center for Analytical Sciences, Tianjin Key Laboratory of Biosensing and Molecular Recognition, College of Chemistry, Nankai University, Tianjin 300071, China.; 2 Department of Intensive Care Unit, Key Laboratory for Critical Care Medicine of the Ministry of Health, Emergency Medicine Research Institute, Tianjin First Center Hospital, School of Medicine, Nankai University, Tianjin 300071, China.; 3 Key Laboratory of Functional Polymer Materials of Ministry of Education, Institute of Polymer Chemistry, College of Chemistry, Nankai University, Tianjin 300071, China.

**Keywords:** multidrug-resistant bacteria, silver nanoagent, backgroundless Raman technology, targeted killing, bacterial imaging

## Abstract

**Purpose:** The growing prevalence of multidrug-resistant (MDR) bacteria makes it clinically urgent to develop an agent able to detect and treat infections simultaneously. Silver has served as a broad-spectrum antimicrobial since ancient times but suffers from major challenges such as moderate antimicrobial activity, nonspecific toxicity, and difficulty to be visualized *in situ*. Here, we propose a new photoactive silver nanoagent that relies on a photosensitizer-triggered cascade reaction to liberate Ag^+^ on bacterial surfaces exclusively, allowing the precise killing of MDR bacteria. Additionally, the AgNP core acts as a backgroundless surface-enhanced Raman scattering (SERS) substrate for imaging the distribution of the nanoagents on bacterial surfaces and monitoring their metabolic dynamics in the infection sites.

**Methods:** In this strategy, the photoactive antibacterial AgNP was decorated with photosensitizers (Chlorin e6, Ce6) and Raman reporter (4-Mercaptobenzonitrile, 4-MB) to provide new opportunities for clinically monitoring and fighting MDR bacterial infections. Upon 655 nm laser activation, the Ce6 molecules produce ROS efficiently, triggering the rapid release of Ag^+^ from the AgNP core to kill bacteria. Poly[4-O-(α-D-glucopyranosyl)-D-glucopyranose] (GP) was introduced as bacteria-specific targeting ligands. SERS spectra of the prepared GP-Ce6/MB-AgNPs were recorded after injecting for 0.5, 4, 8, 12, 24, and 48 h to track the dynamic metabolism of the nanoagents and thus guiding the antibacterial therapy.

**Results:** This new antimicrobial strategy exerts a dramatically enhanced antibacterial activity. The *in vitro* antibacterial efficiencies of this non-antibiotic technique were up to 99.6% against Methicillin-resistant Staphylococcus aureus (MRSA) and 98.8% against Escherichia coli (EC), while the *in vivo* antibacterial efficiencies for MRSA- and Carbapenem-resistant Pseudomonas aeruginosa (CRPA)-infected mice models were 96.8% and 93.6%, respectively. Besides, backgroundless SERS signal intensity of the wound declined to the level of normal tissue until 24 h, indicating that the nanoagents had been completely metabolized from the infected area.

**Conclusion:** Given the backgroundless monitoring ability, high antibacterial efficacy, and low toxicity, the photoactive cascading agents would hold great potential for MDR-bacterial detection and elimination in diverse clinical settings.

## Introduction

Bacterial infections, particularly with the emergence of multidrug-resistant (MDR) bacteria, have become an urgent threat to public health 1, 2. At present, > 700,000 deaths are caused by antimicrobial-resistant bacteria worldwide each year, and this number will reach 10 million per year by 2050 if new antimicrobials are not developed 3-5. Unfortunately, the pipeline of new antibiotics has been declining for several decades, while many currently available antibiotics will no longer be effective against new MDR superbugs 6-8. Therefore, it is highly required to develop alternative antimicrobial therapies to combat MDR infections.

Silver is an intriguing material that has been used as a broad-spectrum antimicrobial since antiquity, although its exact mechanism of action is unclear thus far. 9 So far, studies have reported that silver ions interfere with the normal metabolic process and eliminate bacteria by increasing the permeability of bacterial membranes, hindering nucleic acid replication, inactivating proteins, etc. 10-11. In recent years, the crisis of antibiotic resistance has driven a revival of silver therapy against bacterial infections. For example, silver nanoparticles (AgNPs) are incorporated into dressings 12-14, creams 15, 16 and devices as antimicrobial products 17-20. However, the AgNP-based therapy faces several major challenges that hamper its extensive applications. First, the use of AgNPs alone often shows a moderate antimicrobial effect, especially against acute infections. Second, the AgNP-induced killing is inherently nonspecific, resulting in an inevitable toxicity effect to the host mammalian cells 21-23. More recently, a series of studies have been devoted to enhancing the antimicrobial performance of AgNPs while reducing their nonspecific toxicity. For instance, the endogenous stimuli in the infection microenvironment, such as low pH 24, 25, proteases 26, 27, and glutathione 28, 29 were employed to trigger the release of Ag^+^ *in situ* to exert precision bacteria-killing actions. The antibacterial performance is largely dependent on the bacterial activity, which remains challenging to control on demand. Furthermore, tracking the AgNP-based antimicrobial agents on bacteria is crucial for managing the treatment, but it is difficult to visualize the AgNPs in real time during the therapeutic process.

**Figure 1:**
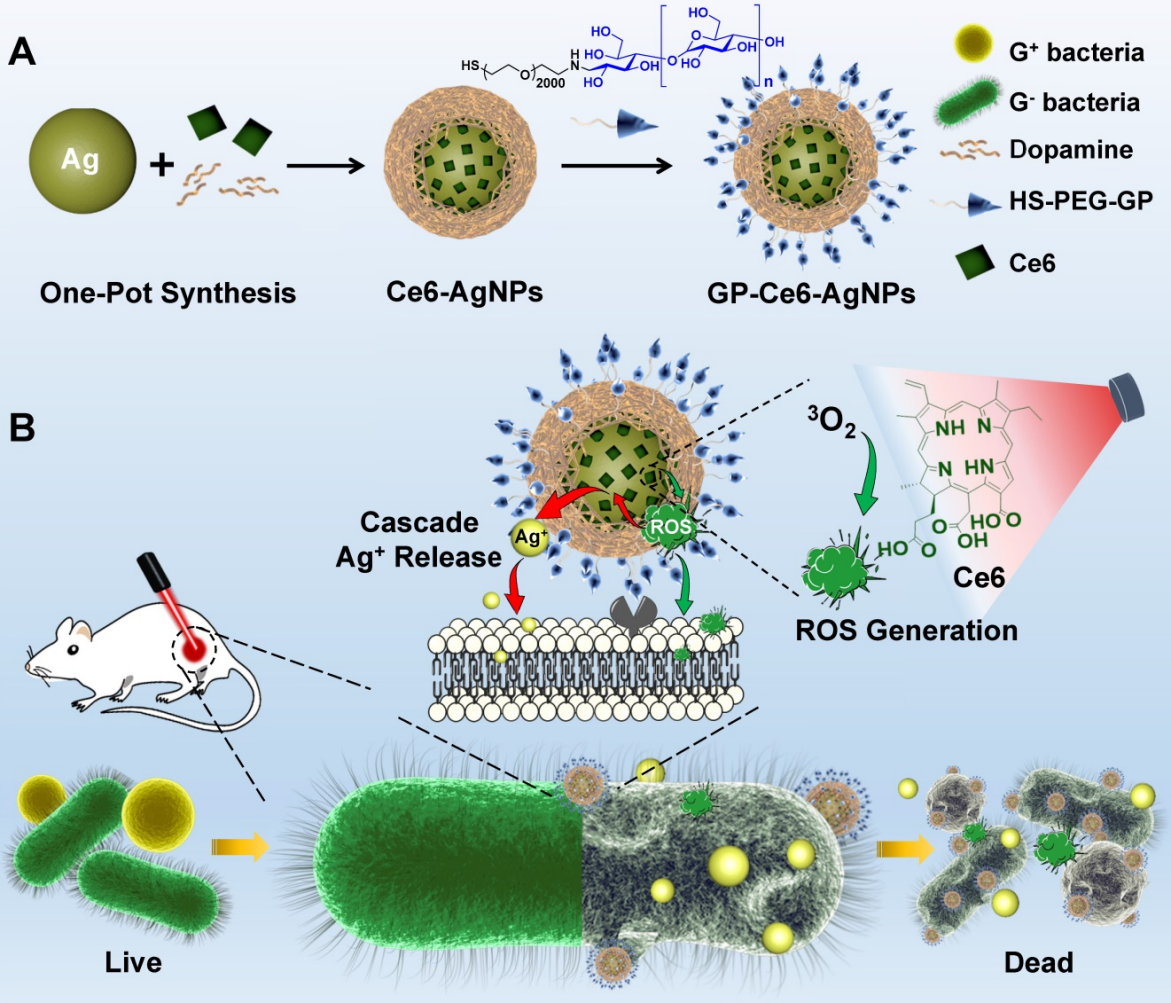
Schematic illustrations of the preparation of GP-Ce6-AgNPs and the GP-Ce6-AgNPs antibacterial mechanism. (**A**) The synthesis route of GP-Ce6-AgNPs. (**B**) Photo-activated cascade release of Ag^+^ for precisely eliminating MDR bacteria. The GP-Ce6-AgNPs specifically target and tightly bind to the surface of bacteria. Upon light activation, the Ce6 molecules efficiently generate ROS, which etches the AgNP core to trigger the release of Ag^+^ *in situ* via a cascade reaction, exerting an on-demand bacterial killing. The excessively generated ROS would take part in the antibacterial action synergistically.

In this study, we report a new class of photoactive silver nanoagents that allow targeted killing of diverse bacteria with high efficacy and specificity. Simultaneously, the nanoagents could be monitored in real-time without background interference both on bacterial cells and in infection sites. This rational design was inspired by the fact that silver can be etched under mild oxidizing conditions, even in the presence of intracellular reactive oxygen species (ROS) 30-32. Based on this, we prepared a photoactive antibacterial AgNP, whose surfaces were decorated with photosensitizers (Chlorin e6, Ce6) and bacteria-targeting ligands via the mussel-inspired chemistry (**Figure 1**) 33, 34. Upon 655 nm laser activation, the Ce6 molecules produce ROS efficiently, triggering the rapid release of Ag^+^ from the AgNP core to kill bacteria. As a bonus of photo-activation, the excess ROS could also involve in the bacteria-killing action via photodynamic therapy (PDT) to further enhance the antibacterial efficacy 35-37. Due to the excellent spatiotemporal resolution of light, this photoactivated strategy achieves on-demand release of Ag^+^ on bacterial surfaces to kill MDR bacteria precisely with dramatically reduced acute and systemic toxicities. In addition, the AgNP core acts as not only an Ag^+^ source but also a typical surface-enhanced Raman scattering (SERS) substrate for imaging the nanoagent-labelled bacteria and further reporting the metabolic dynamics of the nanoagents in the infection sites (**Figure 3**) 38-40.

## Results and Discussion

### Preparation and characterizations of GP-Ce6-AgNPs

The light-activated antibacterial nanoagents were synthesized by a mussel-inspired synthesis strategy. Under a weak alkaline condition (pH 8.5), dopamine is able to form a nest-like polydopamine (PDA) shell onto AgNPs, by which Ce6 dyes were incorporated into the surface-adherent shells to form Ce6-functionalized AgNPs (Ce6-AgNPs). Next, the bacteria-specific targeting ligands, poly[4-O-(α-D-glucopyranosyl)-D-glucopyranose] (GP) 41-44 were tethered on the PDA-coating Ce6-AgNPs by a polyethylene glycol (PEG) linker terminated in amine and thiol on both sides. The amine side was conjugated with the aldehyde of GP via Schiff base reaction,^45^ while the thiol side was labeled to the exposed quinone on the PDA shells via Michael addition reaction 46, 47. As a result, the GP-tagged Ce6-AgNPs (GP-Ce6-AgNPs) were obtained.

**Figure 2:**
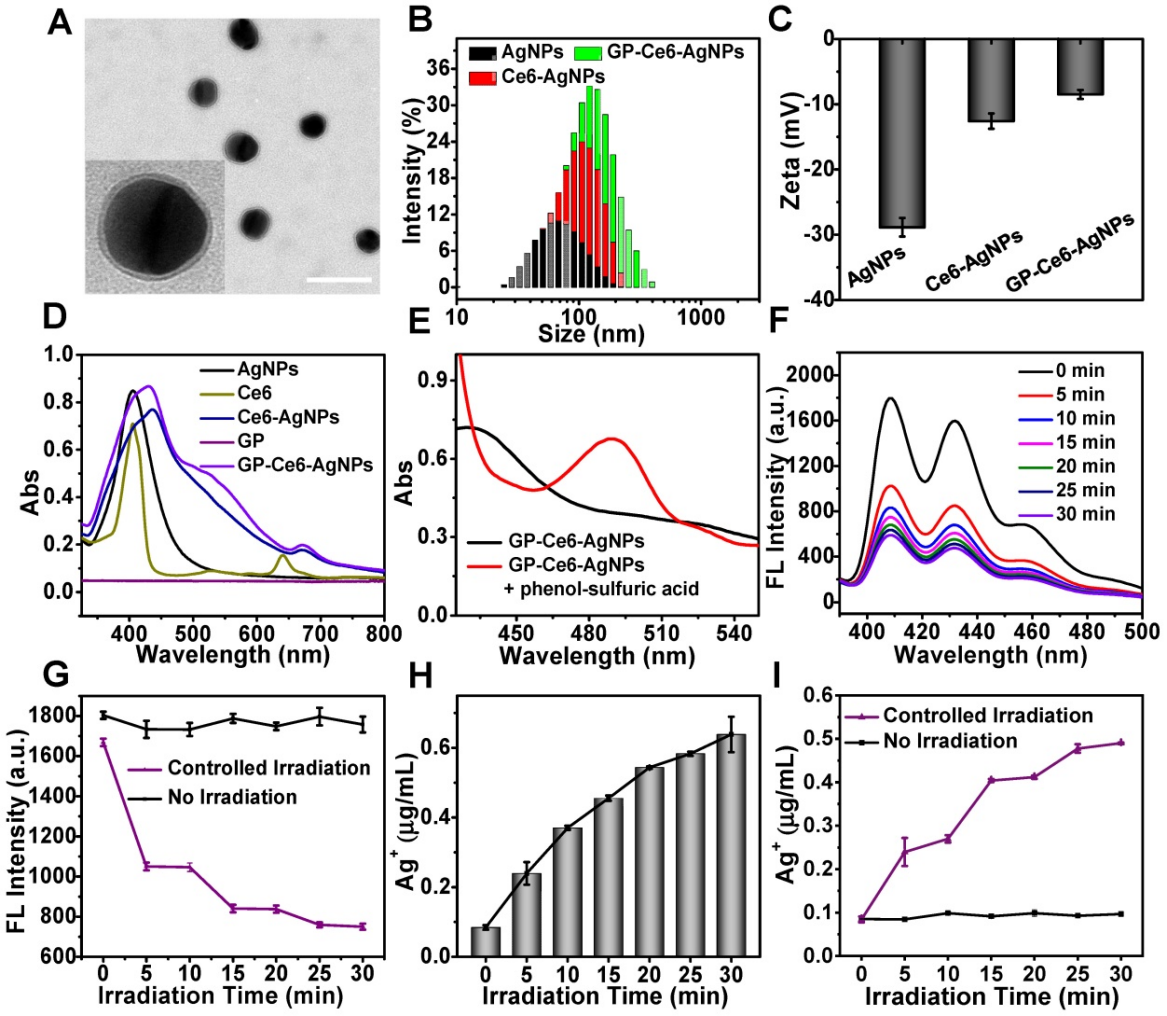
Characterization of GP-Ce6-AgNPs. (**A**) TEM image of GP-Ce6-AgNPs. Scale bar: 100 nm. (**B**) DLS spectra of AgNPs, Ce6-AgNPs, GP-Ce6-AgNPs. (**C**) Zeta potentials of AgNPs, Ce6-AgNPs, GP-Ce6-AgNPs. (**D**) UV-Vis absorbance of AgNPs, Ce6, Ce6-AgNPs, GP, GP-Ce6-AgNPs. (**E**) UV-Vis absorbance of GP-Ce6-AgNPs and GP-Ce6-AgNPs treated with phenol-sulfuric acid. The absorption peak at around 490 nm indicated that the GP molecules were successfully linked to Ce6-AgNPs. (**F**) ROS generation abilities of GP-Ce6-AgNPs. (ABDA worked as the ^1^O_2_ indicator) (**G**) Fluorescence intensity of GP-Ce6-AgNPs treated ABDA (*λ*ex = 408 nm) after exposure to three-cycle light irradiation (purple, 5 min for each irradiation) and no irradiation (black). (**H**) Light-activated Ag^+^ release ability determined by ICP-OES. (**I**) The amount of Ag^+^ after exposure to three-cycle light irradiation (purple, 5 min for each irradiation) and no irradiation (black). All experiments in (**F**-**H**) were performed with a 655 nm laser irradiation (300 mW/cm^2^).

As observed from the transmission electron microscope (TEM, **Figure 2A**), the GP-Ce6-AgNPs show a well-defined core-shell spherical structure with an average diameter of 49.4 ± 4.4 nm and a PDA shell of 3.6 ± 0.6 nm in thickness. The hydrodynamic diameter of the GP-Ce6-AgNPs was determined to be ~160 nm by dynamic light scattering (DLS) (**Figure 2B**), much larger than the size measured by TEM. The dramatically increased DLS value over actual particle size could be attributed to the thick hydration shell resulted from the abundant hydroxyl groups of GP surrounding water molecules. With the coating of PDA and GP, the particles became less negatively-charged gradually (**Figure 2C**). The preparation processes were further monitored by UV-Vis spectroscopy. As depicted in **Figure 2D**, the GP-Ce6-AgNPs exhibited two remarkable absorption peaks at around 431 and 670 nm. The former peak could be characteristic of AgNPs; the latter one indicates the loaded Ce6. Compared to the peaks of the naked AgNPs and free Ce6, the two peaks of GP-Ce6-AgNPs red-shifted markedly, most likely owing to the PDA coating and Ce6 stacking on the AgNP surfaces respectively.

A phenol-sulfuric acid assay, the most classical method for carbohydrate analysis, was used to analyze the tagged GP 48. This colorimetric assay is based on the reactions between polysaccharide, sulfuric acid, and phenol, which produce a final orange-yellow compound exhibiting a typical peak at approximately 490 nm. After treating the GP-Ce6-AgNPs with sulfuric acid and phenol in sequence, we observed a new peak at 490 nm (**Figure 2E** and **Figure S1**), indicating the presence of GP on the particles. We attempted to quantify the amounts of GP and Ce6 on each AgNP. With the aid of phenol-sulfuric acid assay, we have created two calibration curves by plotting the absorbance at 490 nm and 645 nm with various concentrations of free GP and Ce6, respectively (**Figure S2**), by which the amounts of the loaded GP and Ce6 on each GP-Ce6-AgNP were quantified to be 2.9 × 10^4^ and 5.7 × 10^4^ respectively (see details in the Experimental Section).

### Light-activated release of Ag^+^ from GP-Ce6-AgNPs

With the GP-Ce6-AgNPs in hand, we next investigated the photo-activated release of ROS and Ag^+^ using different assays. The light-activated ROS from the GP-Ce6-AgNPs was measured by 9,10-anthracenediyl-bis(methylene) dimalonic acid (ABDA) testing. ABDA is commonly used as a ^1^O_2_ indicator, as its anthracene moiety could quickly trap ^1^O_2_ to cause fluorescence bleaching 49. The fluorescent decay of ABDA can thus be employed to quantify ROS. As anticipated, when the mixture of GP-Ce6-AgNPs (30 μg/mL) and ABDA (1 μM) was irradiated by a 655 nm laser, the fluorescence from 395 to 480 nm decreased gradually with the irradiation time (**Figure 2F** and **Figure S3**), indicating the generation of ROS.

To further test the controllability, the mixture of GP-Ce6-AgNPs and ABDA was exposed to the laser with the light on and off alternatively. The other group without the laser irradiation was set as a control. As shown in **Figure 2G**, once the mixture was irradiated for 5 min, the fluorescence intensity at 408 nm decreased dramatically. When the laser is turned off for another 5 min, the fluorescence intensity remains unchangeable in the dark. The photo-activated processes can be repeated for at least three cycles. In contrast, if the mixture was not exposed to laser irradiation, the fluorescence quenching cannot be found during the same period. This result reveals that the GP-Ce6-AgNPs could efficiently generate ROS under 655 laser irradiation for silver etching, which can be activated on demand by a photo-switcher. In parallel, the amounts of Ag^+^ released from the GP-Ce6-AgNPs were recorded by an inductively coupled plasma optical emission spectrometer (ICP-OES). When the mixture of GP-Ce6-AgNPs was treated with continuous irradiation for 30 min, a graduate release of Ag^+^ was detected (**Figure 2H**). Once the sample was exposed to the laser with the light on and off alternatively, a remarkable release of Ag^+^ can be observed within the first 5 min (on), and the amount of Ag^+^ reached nearly constant from 5 to 10 min (off), followed by a similar pattern in the other two irradiation cycles (**Figure 2I**). The trend of Ag^+^ release corresponded perfectly to that of ROS generation, which was attributed to a cascade reaction. The loaded Ce6 trigged the conversion of ^3^O_2_ into ROS (typically ^1^O_2_), which shows an etching effect toward the AgNP core, giving rise to accelerated release of Ag^+^. Once the illumination was switched off, the Ag^+^ generation was paused immediately. Moreover, it was worthwhile that the release of Ag^+^ was gradually enhanced with the increasing laser power density (**Figure S4**). The results demonstrate the excellent controllability of light to release Ag^+^, showing great potential for antibacterial applications.

### Bacterial Targeting, Imaging, and Monitoring

**Figure 3:**
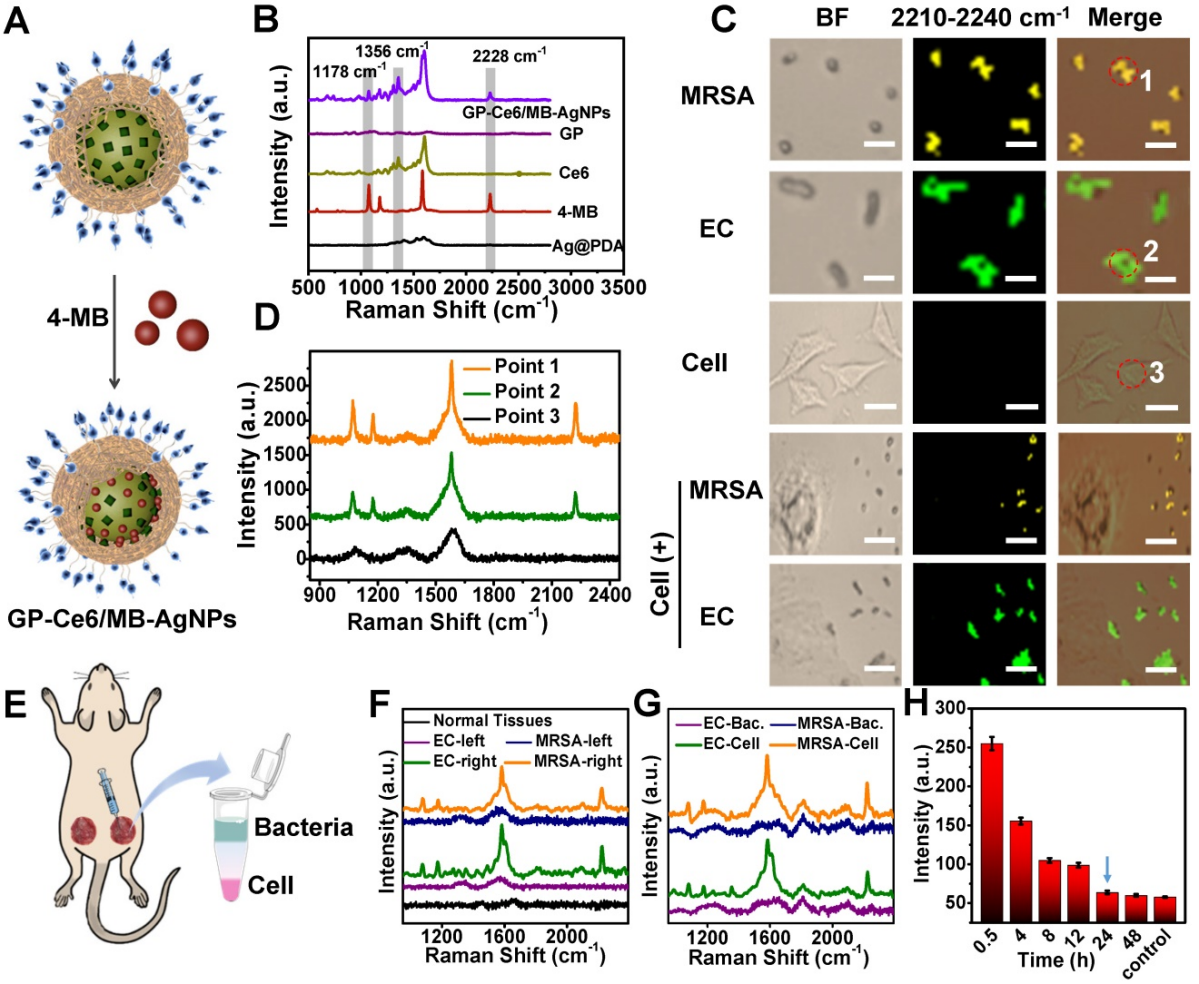
Backgroundless Raman imaging and tracking of GP-Ce6/MB-AgNPs. (**A**) Schematic illustration of GP-Ce6/MB-AgNPs. 4-MB was introduced on the surface of AgNPs during the PDA polymerization process. (**B**) Raman spectra of Ag@PDA, 4-MB, Ce6, GP, GP-Ce6-AgNPs recorded by a confocal Raman microscope (Renishaw) with 532 nm He-Ne laser (30 mW, 1 s). (**C**) Raman mapping images in the nitrile channel (2228 cm^-1^) for GP-Ce6/MB-AgNPs on MRSA, EC, 3T3 cell, MRSA + 3T3 cell, and EC + 3T3 cell by a confocal Raman microscope with 532 nm laser (30 mW, 2 s). Scale bar: the top two rows, 2 μm; the middle row, 10 μm; the bottom two rows: 5 μm. Pseudo-colors yellow and green represent the signals of MRSA and EC, respectively. (**D**) SERS spectra obtained from Points 1, 2, and 3 in (**B**). (**E**) Schematic illustration of *in vivo* monitoring of GP-Ce6/MB-AgNPs. The two wounds on the thighs of a Balb/c mouse were first infected by bacteria suspensions of MRSA and EC; the right wound was then injected with 100 μL of GP-Ce6/MB-AgNPs (30 μg/mL) while the left one was injected with PBS as a control. The harvested tissues at the wound area were treated with a collagenase hydrolysis method to separate bacteria and cells. (**F**) Raman spectra were obtained from the infectious wound areas after incubating with GP-Ce6/MB-AgNPs for 1 h. (**G**) Raman spectra of the separated layers of bacteria and cells. (**H**) Raman signals at 2228 cm^-1^ (intensity per mm^2^) for the wound area treated with GP-Ce6/MB-AgNPs over time. Raman spectra of (**F**-**H**) were collected with a confocal Raman microscope (Renishaw) with 532 nm He-Ne laser (30 mW), 5× objective lens, 50 × 50 steps, and an exposure time of 2 s.

It is vital to visualize and track the antibacterial agents on targeted bacteria in various environments. Besides as a Ag^+^ source, the AgNP core also acts as a typical SERS substrate for exploring the targeting property of the nanoagents and monitoring their distribution on bacteria cells 38-40. 4-Mercaptobenzonitrile (4-MB), a background-free Raman reporter, was introduced to the surface of AgNPs to yield 4-MB co-loaded GP-Ce6-AgNPs (GP-Ce6/MB-AgNPs) (**Figure 3A**). From the Raman spectra in **Figure 3B**, the GP-Ce6/MB-AgNPs displayed three characteristic single peaks at 1356 (assigned to Ce6), 1178, and 2228 cm^-1^ (assigned to 4-MB), suggesting the successful fabrication of the theranostic nanoagents. Other groups and we have demonstrated that the single sharp peak at 2228 cm^-1^ appears in the biological Raman-silent (term as backgroundless) window (1800-2800 cm^-1^), which can be employed for Raman imaging 50, 51. Compared with Raman peaks from 1000-2000 cm^-1^ of biomolecules, the peak in Raman-silent window has no background interference, thus make it more reliable to monitor the dynamic changes of Raman probe. Before bringing the GP-Ce6/MB-AgNPs into bacteria imaging, their SERS stability under laser irradiation was investigated. As depicted in **Figure S5**, the Raman signals at 2228 cm^-1^ had slightly changed with or without laser irradiation for 30 min, indicating the excellent chemical and colloidal stability of the resulted GP-Ce6/MB-AgNPs.

Next, the targeting performance was investigated by Raman imaging in the 2228 cm^-1^ channel. Methicillin-resistant *Staphylococcus aureus* (MRSA, a typical Gram-positive bacteria) and *Escherichia coli* (EC, a typical Gram-negative bacteria) were incubated with the GP-Ce6/MB-AgNPs respectively at 37°C for 1 h. Then, 1 μL of the rinsed bacterial suspension was dropped on a glass slide for Raman imaging under a confocal Raman microscope. As shown in **Figure S6**, the nitrile signals from the GP-Ce6/MB-AgNPs are largely distributed on the bacteria. The yellow and green pseudo-colors represented the signals from the GP-Ce6/MB-AgNP-stained MRSA and EC, respectively.

Furthermore, the distribution of GP-Ce6/MB-AgNPs on bacteria surfaces can be mapped at single-cell levels. Based on the background-free Raman imaging technique, we investigated the selective affinity of the nanoagents to bacteria. GP can recognize bacteria exclusively through GP-mediated transporter that is expressed on bacteria surfaces but not mammalian cells. To validate this, the same concentration of GP-Ce6/MB-AgNPs were incubated with mammalian 3T3 cells and those mixed with MRSA and EC, respectively. As shown in **Figure 3C**, no Raman signals of the nanoagents can be found on the 3T3 cells. Even for the two mixtures of MRSA + 3T3 and EC + 3T3, the nitrile signals can only be observed on the bacteria. **Figure 3D** reveals the relevant Raman spectra of the points shown in **Figure 3C**, confirming the high accuracy of the imaging results. The Raman imaging results indicate high specificity and targeting of the nanoagents to both gram-positive and gram-negative bacteria.

The *in vivo* targeting ability of GP-Ce6/MB-AgNPs to bacteria was evaluated by building two wounds on the thighs of a Balb/c mouse. The right wound was injected with 100 μL of GP-Ce6/MB-AgNPs (30 μg/mL) while the left one was injected with PBS as a control (**Figure 3E**). The Raman spectra were recorded *in situ* (**Figure 3F**). Obviously, the nanoagent-treated infected wound exhibited a distinct nitrile peak at 2228 cm^-1^, whereas no characteristic signals were detected from normal tissues and the infected tissues treated with PBS. To verify whether the Raman signals originated from the bacteria or the mammalian cells at the infected wound, a collagenase hydrolysis method was employed to analyze the harvested wound tissue. In short, the obtained tissue was enzymatically hydrolyzed into a homogenous solution 52, where the bacteria and mammalian cells were separated using a density-gradient centrifugation method. The Raman spectra demonstrated that almost all the signals at 2228 cm^-1^ originated from the bacteria (**Figure 3G**), confirming the outstanding targeting ability of GP-Ce6/MB-AgNPs to bacteria.

Encouraged by these positive results, we monitored the metabolic dynamics of GP-Ce6/MB-AgNPs in the infected wound *in situ* after injecting for 0.5, 4, 8, 12, 24, and 48 h. More than 1000 spectra were recorded at each time point. **Figure 3H** illustrated that the average signal intensity of nitrile decreased over time. Until 24 h, the signal intensity of the wound declined to the level of normal tissue, indicating that the nanoagents had been completely metabolized from the infected area. The backgroundless Raman imaging technique outperforms conventional methods (typically mass spectroscopy) for tracking the dynamic metabolism of the nanoagents and thus guiding the antibacterial therapy.

### *In vitro* antibacterial effect and mechanism investigation

**Figure 4:**
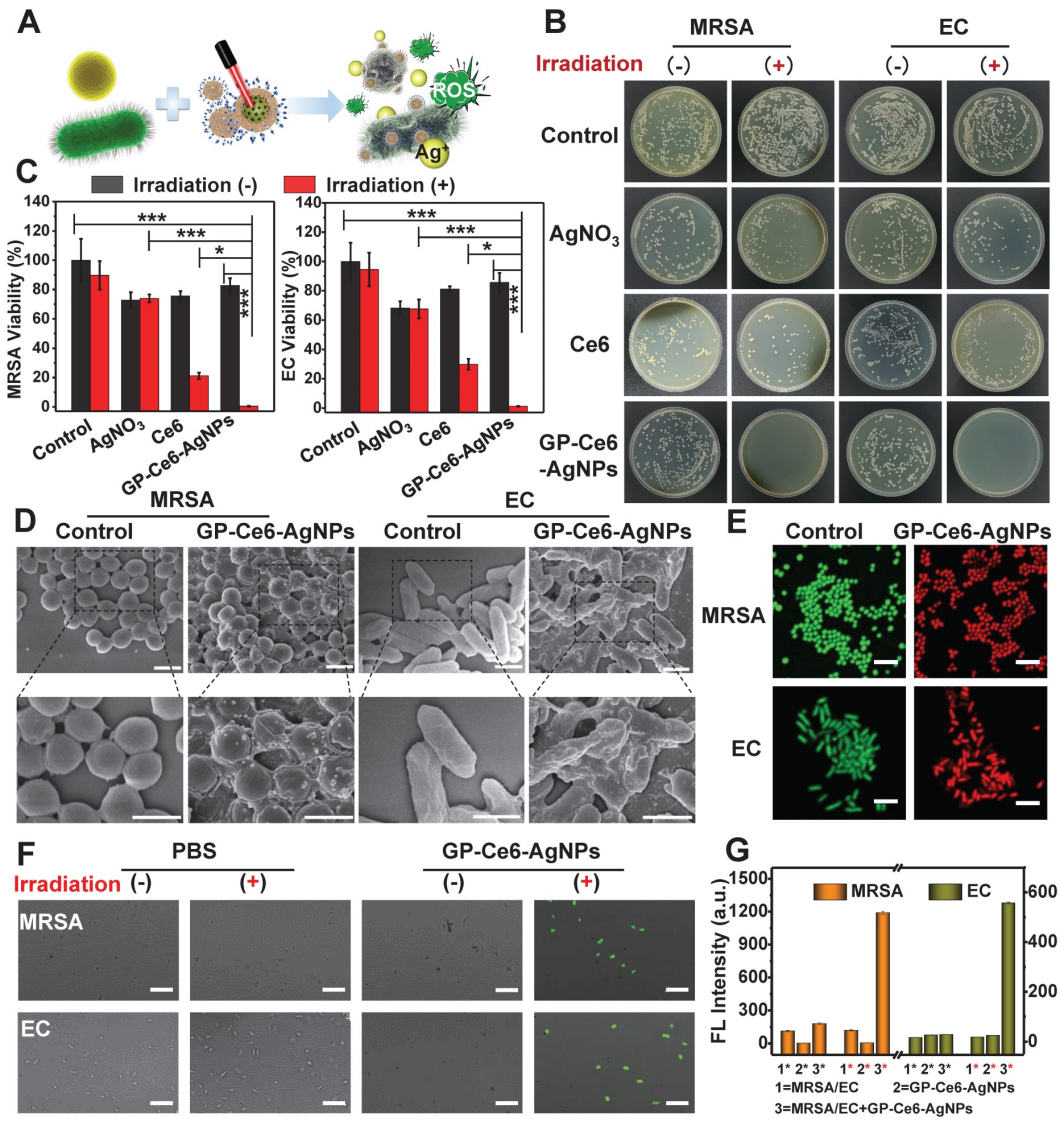
*In vitro* antibacterial performance of GP-Ce6-AgNPs. (**A**) Schematic illustration of the antibacterial mechanism of GP-Ce6-AgNPs. (**B**) Photographs of the agar plates of MRSA and EC treated with PBS (control), AgNO_3_ (2 μg/mL), Ce6 (200 μM), and GP-Ce6-AgNPs (30 μg/mL) treated with and without constant 655 nm laser irradiation espectively. (**C**) The corresponding bacterial viability in panel (**B**). (**D**) SEM images of MRSA and EC incubated with PBS (control) and GP-Ce6-AgNPs after constant irradiation. Scale bar: 1 μm. (**E**) Representative CLSM images for a live/dead bacterial staining assay of MRSA and EC with PBS (control) and GP-Ce6-AgNPs after constant irradiation. (meilungreen: *λ*ex = 486 nm, *λ*em = 525; PI: *λ*ex = 561 nm, *λ*em = 595 nm). Scale bar: 5 μm. (**F**) Measurement of ROS level in MRSA and EC using CLSM with DCFH-DA staining (*λ*ex = 486 nm, *λ*em = 525 nm). Scale bar: 10 µm. (**G**) FL intensity of MRSA, GP-Ce6-AgNPs, MRSA + GP-Ce6-AgNPs, EC, GP-Ce6-AgNPs, and EC + GP-Ce6-AgNPs with and without irradiation by using DCFH-DA fluorescence method, respectively. The red * represented groups treated with 655 nm irradiation (10 min, 300 mW/cm^2^) while the black * represented no irradiation treatment. All experiments were performed with a 655 nm laser irradiation (10 min, 300 mW/cm^2^). P-value (*: P < 0.05; **: P < 0.01; ***: P < 0.001).

After demonstrating the high targeting ability of GP-Ce6-AgNPs to bacteria, we investigated their efficacy in killing diverse bacteria. MRSA and EC, the representative gram-positive and gram-negative bacteria, were selected to estimate the photo-activated antibacterial activity of GP-Ce6-AgNPs (**Figure 4A**). The standard colony counting was first performed to evaluate the antibacterial efficiency of GP-Ce6-AgNPs, AgNO_3_, and free Ce6 under the same conditions. The amounts of Ag in AgNO_3_ and free Ce6 were identified to those of the loaded Ag and Ce6 in GP-Ce6-AgNPs respectively. The bacteria treated with PBS alone were set as controls. As depicted in **Figure 4B**, both MRSA and EC showed numerous bacterial colonies in the control groups. With the treatment of AgNO_3_, the bacterial counts reduced partly compared to the control groups. However, light irradiation has a negligible impact on the antibacterial effects of AgNO_3_. Ce6, the most common photosensitizer, showed a moderate efficacy to kill bacteria under 655 nm laser irradiation for 10 min. By contrast, the proposed GP-Ce6-AgNPs could eliminate both MRSA and EC with strikingly high efficacy under the same light irradiation conditions. The antibacterial efficacies can be quantified by counting the bacterial colonies using Image J software. The relative antibacterial rates of the light-activated GP-Ce6-AgNPs were estimated to be 99.6% against MRSA and 98.8% against EC (**Figure 4C**, see detailed data in **Table S1**).

The antibacterial mechanism of GP-Ce6-AgNPs was systematically explored by scanning electron microscope (SEM) imaging, live/dead bacterial staining, and 2,7-dichlorodihydrouorescein diacetate (DCFH-DA) fluorescent imaging. As depicted in **Figure 4D**, GP-Ce6-AgNPs are distributed on the bacterial surfaces, which intuitively supported the remarkable targeting ability of GP-Ce6-AgNPs. Prior to light irradiation, the bacteria displayed intact morphology and smooth cell walls. Once irradiated by 655 nm light (300 mW, 10 min), the cell walls became rough and wrinkled, combined with lysed debris. The SEM results preliminarily suggest that the inactivation mechanism can be attributed to the disruption of bacterial membrane.

A live/dead bacterial staining assay was further performed to investigate the membrane permeability of bacteria. When the nanoagent-treated bacteria were incubated with the mixture of meilungreen (a live-cell staining dye) and propidium iodide (PI, a dead-cell staining dye), the bacteria with intact cell membranes exhibited bright green fluorescence while those with damaged membranes exhibited strong red fluoresence. **Figure 4E** revealed that nearly all bacteria in the control groups emitted the green fluorescence of meilungreen. The bacteria treated with GP-Ce6-AgNPs followed by a continuous 10 min irradiation showed the typical red fluorescence of PI. The results confirmed the markedly enhanced permeability of bacterial membranes during the photo-activated antibacterial process.

The antibacterial mechanism was also supported by the 2,7-dichlorodihydrouorescein diacetate (DCFH-DA) fluorescent assay. Production of hydroxyl radicals (OH•) is widely recognized as a common mechanism of bacteria death, although its concrete role is still in debate 53. DCFH-DA, which has no fluorescence itself, could freely penetrate through the bacterial membrane to react with the esterase to yield DCFH inside the bacteria 54. Once exposed to OH•, the DCFH can be oxidized to generate fluorescent DCF. As a result, the fluorescence of the detected DCF could be employed to evaluate the viability of bacteria. From the confocal laser scanning microscope (CLSM) images (**Figure 4F**), only the GP-Ce6-AgNP-treated bacteria (followed by 633 laser irradiation) showed intense DCF fluorescence, while the other groups remained fluorescently silent. The generated OH• might be attributed to the excessive oxidative stress when bacteria suffered from the agonal stage. **Figure 4G** showed the fluorescence intensity of DCF in MRSA and EC under various treatments before and after laser irradiation.

On the basis of the above investigations, the light-activated antibacterial mechanism of GP-Ce6-AgNPs can be reasoned as follows. First, the GP-Ce6-AgNPs recognized the ATP-binding cassette transporters that are specifically expressed on the bacterial surfaces. Then, light irradiation triggered the release of Ag^+^ from GP-Ce6-AgNPs, which disrupted the disulfide bond of proteins to affect the membrane permeability. Additionally, the released Ag^+^ tended to penetrate bacterial membrane to interact with the intracellular proteins and DNA, inducing oxidative damage. As a bonus, the excessively generated ^1^O_2_ may serve as a typical ROS of PDT agent to further enhance the antibacterial efficacy.

### *In vivo* antibacterial effect

**Figure 5:**
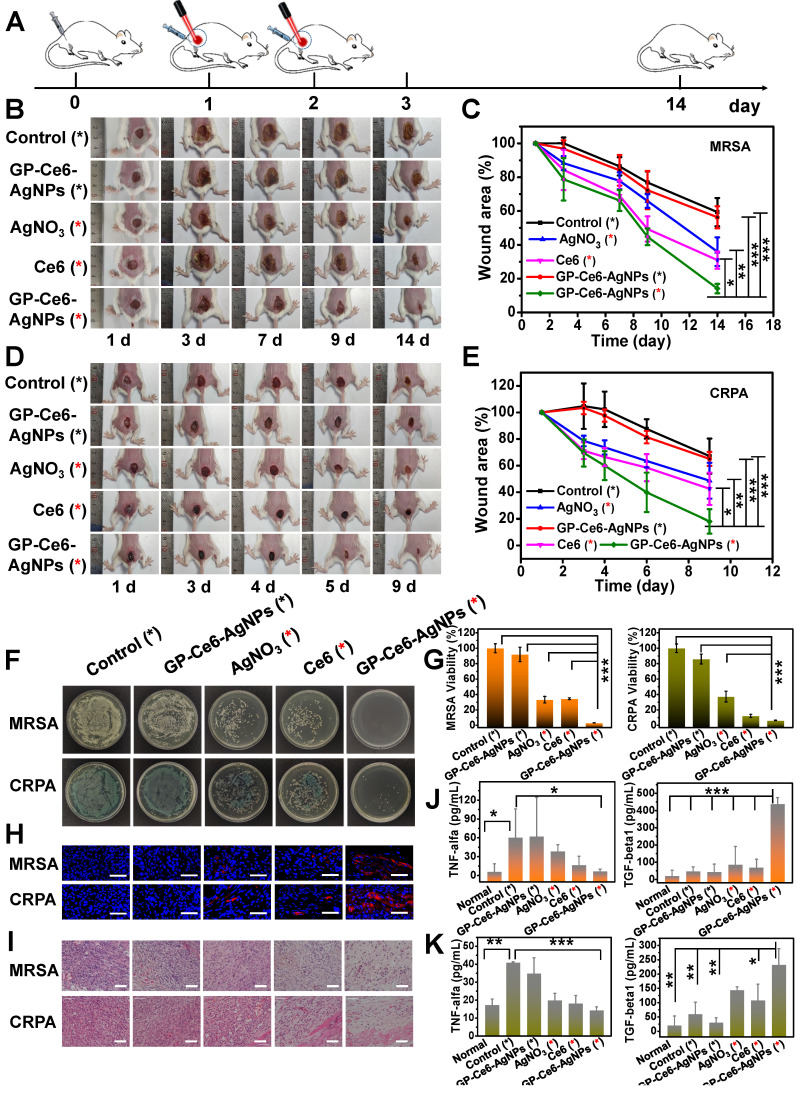
*In vivo* antibacterial performance of GP-Ce6-AgNPs. (**A**) Schematic illustration of the antibacterial treatment procedure in MDR bacteria-infected Balb/c mice model. (**B**) Representative photographs of MRSA-infected mice model treated with PBS (control), AgNO_3_ (2 μg/mL), Ce6 (200 μM) and GP-Ce6-AgNPs (30 μg/mL). The red * represented groups treated with 655 nm irradiation (10 min, 300 mW/cm^2^) while the black * represented no irradiation treatment. (**C**) The corresponding wound area (S/S_0_) of MRSA-infected mice following the treatment procedure. CRPA-infected mice were presented in (**D**) and (**E**). (**F**) Photographs of the agar plates of MRSA and CRPA were collected from the infected wound at 14 days and 9 days, respectively. (**G**) The relative bacterial survival of MRSA and CRPA infected wounds with different treatments (Orange, MRSA; green, CRPA). (**H**) Immunofluorescence images of the wound tissues with different treatments (blue, nucleus; red, VEGF). Scale bar: 50 µm. (**I**) H&E staining images of the wound tissues with different treatments. Scale bar: 5 µm. The levels of pro-inflammatory cytokines (TNF-α) and anti-inflammatory cytokines (TGF-β1) in serum for (**J**) MRSA- and (**K**) CRPA-infected mice (Orange, MRSA; green, CRPA). P-value (*: P < 0.05; **: P < 0.01; ***: P < 0.001).

Inspired by their outstanding targeting and antibacterial activity *in vitro*, GP-Ce6-AgNPs were further brought into *in vivo* antibacterial examinations. MRSA and carbapenem-resistant *Pseudomonas aeruginosa* (CRPA), the most formidable gram-positive and gram-negative MDR superbug in the clinic, were used to build skin wound models on the thighs of a Balb/c mouse. The infected mice were divided into five groups with different treatments. As instructed by the metabolism results of GP-Ce6-AgNPs in **Figure 3H**, the administration interval was set as 24 h, followed by 655 nm laser irradiation (300 mW/cm^2^, 10 min). Both the wound area and bodyweight of the mice were monitored during the whole therapeutic process (**Figure 5A**). The wound areas of bacteria-infected mice were photographed every day, and the relative wound areas (S/S_0_) were measured by Image J software. As shown in **Figure 5B-5C**, the S/S_0_ of GP-Ce6-AgNPs plus irradiation in the MRSA group declined to 14%, while those of GP-Ce6-AgNPs without irradiation, AgNO_3_ plus irradiation, Ce6 plus irradiation, and the control group decreased to 56%, 36%, 31%, 59% respectively. Meanwhile, a similar tendency of S/S_0_ was found in the CRPA-infected mice (**Figure 5D-5E**, and **Table S2**). Notably, the GP-Ce6-AgNPs plus irradiation group can strikingly accelerate the healing of MDR-infected wounds. During the whole treatment process, the bodyweight of the mice had no significant difference (**Figure S7**), indicating excellent biocompatibility of the new therapeutic strategy.

To further assess the antibacterial performance, the mice were sacrificed on the last day of treatment, and the infected tissues, major organs, and sera were harvested for further analysis. After treatment with GP-Ce6-AgNPs plus irradiation, the amounts of bacteria left in the infected tissues were significantly reduced in comparison to those of other groups (**Figure 5F**). The relative antibacterial rates of the light-activated GP-Ce6-AgNPs were processed as 96.8% against MRSA and 93.6% against CRPA, both of which were much higher than the other treatments (**Figure 5G**, and**Table S3**).

Furthermore, we evaluated the wound healing process from the histological aspect with immunofluorescence staining and hematoxylin-eosin (H&E) staining assays. Wound healing was mainly related to inflammation, proliferation, and tissue remodeling 55, 56. We first measured the expression of VEGF in the wound tissues because VEGF plays a central role in promoting wound healing 57, 58. As shown in **Figure 5H**, the infected wounds treated with GP-Ce6-AgNPs plus irradiation exhibited the highest VEGF expression levels compared with other groups. The H&E staining images (**Figure 5I**) show that numerous inflammatory cells, including neutrophils and mononuclear cells, can be clearly found in the PBS-only control group, while the infiltration of inflammatory cells decreased to certain extents after treating with AgNO_3_ plus irradiation and Ce6 plus irradiation. By contrast, negligible inflammatory cells can be observed in the infected tissues after treatment with GP-Ce6-AgNPs plus irradiation, whereas obvious re-epithelialization and fibroblasts were found in the same tissues. These results suggest the high activity of the light-activated GP-Ce6-AgNPs in accelerating wound repair.

To better understand the inflammatory response in wound healing, we employed enzyme-linked immunosorbent assays (ELISA) to determine the levels of inflammatory factors including mouse pro-inflammatory cytokines (TNF-α) and anti-inflammatory cytokines (TGF-β1) in sera (**Figure 5J-5K**) 59. For the PBS-only control group, the infected mice posed a high level of TNF-α in their serum, suggesting an excessive inflammatory response that is harmful to wound repair. For the AgNO_3_ plus irradiation and Ce6 plus irradiation groups, the levels of TNF-α decreased slightly. With the treatment of GP-Ce6-AgNPs plus irradiation, the TNF-α level in the infected serum decreased to the level in normal serum without bacterial infection. Meanwhile, the GP-Ce6-AgNPs plus irradiation group showed a significantly higher expression level of TGF-β1 than the other groups. It is generally recognized that TGF-β1 stimulates fibroblast differentiation into myofibroblasts to promote the formation of new capillary, extracellular matrix, and collagen, which plays an important role in wound repair at the late infection stages 60.

### Biosafety evaluation of GP-Ce6-AgNPs

**Figure 6:**
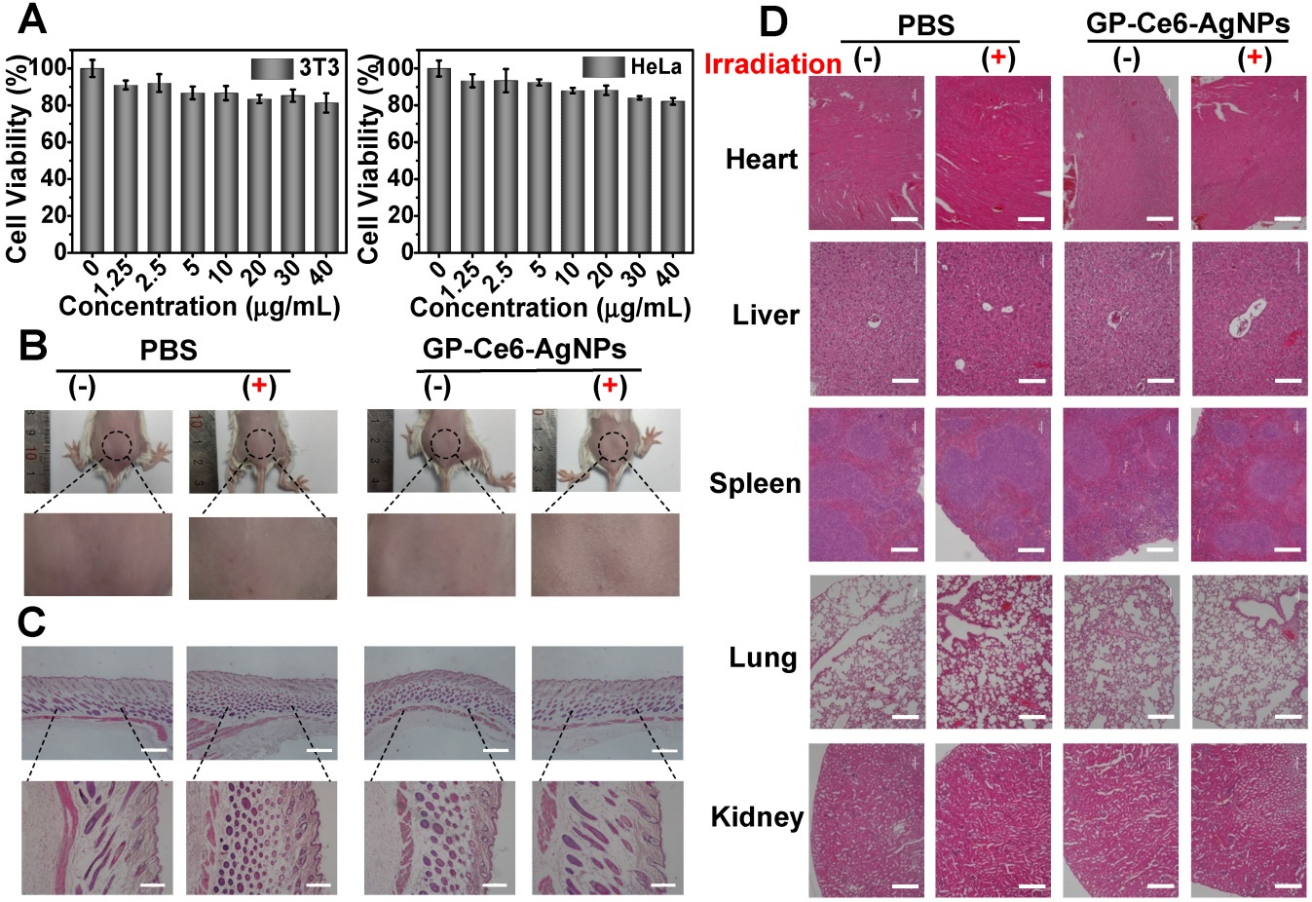
Biological safety evaluation of GP-Ce6-AgNPs. (A) Cell viability of 3T3 and HeLa cells treated with different dosages of GP-Ce6-AgNPs (0, 1.25, 2.5, 5, 10, 20, 30, 40 μg/mL) by MTT assay. (B) Photograph of skin tissues at 14 Day after injection with PBS (control) and GP-Ce6-AgNPs (100 μL, 30 mg/mL) on healthy mice and then treatment without and with constant 655 nm irradiation (10 min, 300 mW/cm^2^). (C) H&E staining images of skin tissues with different treatments. Scale bar: the top row, 500 µm; the bottom row, 200 µm. (D) H&E staining images of the major organs (heart, liver, spleen, lung, and kidney) from the model mice with different treatments. Scale bar: the second row (liver), 100 µm; others, 200 µm.

Aside from antibacterial efficacy, biosafety is another crucial parameter for the practical applications of therapeutic agents. The *in vitro* biosafety was first checked by methyl thiazolyl tetrazolium (MTT) assays. As depicted in **Figure 6A**, the viability of both 3T3 fibroblast cells and HeLa cells remained above 85% even when the concentration of GP-Ce6-AgNPs went up to 30 µg/mL.

To further evaluate the biosafety *in vivo*, 100 μL of GP-Ce6-AgNPs (30 μg/mL) were injected into the subcutaneous tissue of healthy mice, followed with or without a 655 nm laser irradiation (10 min, 300 mW/cm^2^). The injected skin was analyzed after the respective treatment for 14 days. The photographs in **Figure 6B** show that the skin surfaces at the injection site were considerably smooth, and no wrinkles and scars were observed in the skin. The histological analysis of skin tissues demonstrated no significant difference between GP-Ce6-AgNPs and PBS (control) treatments under both dark and illumination conditions (**Figure 6C**). Furthermore, the main organs including heart, liver, spleen, lung, and kidney were resected from the GP-Ce6-AgNP- and PBS-treated healthy mice after 14-day treatment with or without irradiation. Through analysis of the histological results, normal morphological features were detected in the biopsy sections of all resected organs (**Figure 6D**), indicating excellent biocompatibility of the photo-activated GP-Ce6-AgNPs. All these results strongly support that the newly developed therapeutic agents could be clinically employed as an effective and safe platform to eradicate MDR infections.

## Conclusions

In this study, we have developed a photoactive imaging and antibacterial nanoagent to monitor and kill diverse MDR bacteria with high precision and efficacy. This new antimicrobial strategy relies on the photo-controlled release of Ag^+^, which exerts a dramatically enhanced antibacterial effect against MDR bacteria. The *in vitro* antibacterial efficiencies of this non-antibiotic technique were up to 99.6% against MRSA and 98.8% against EC, while the *in vivo* antibacterial efficiencies for MRSA- and CRPA-infected mice models were 96.8% and 93.6%, respectively. Such high antibacterial performance of the nanoagents could be attributed to the excellent bacteria-specific targeting, spatiotemporal controllability of light, sustained killing performance of Ag^+^, and a supplementary antibacterial effect of ROS. Besides, incorporation of exogenous Raman dyes on the antibacterial nanoagents allows backgroundless imaging and real-time monitoring of the nanoagents' metabolic dynamics in the infection sites. Given the backgroundless monitoring ability, high antibacterial efficacy, and low toxicity, the photoactive cascading agents would hold great potential for MDR-bacterial detection and elimination in diverse clinical settings.

## Experimental Section

### Preparation of silver nanoparticles (AgNPs)

AgNPs were synthesized according to our group's work with a slight modification 61. Firstly, all glassware was washed with freshly prepared HNO_3_-HCl (3:1 v/v) before use, then rinsed carefully with deionized water. Briefly, 150 μL of the aqueous L-ascorbic acid (1 M) was hereby added into 300 mL of boiling water, with a final concentration of 0.5 mM while stirring vigorously. Then, 6 mL of an aqueous solution of sodium citrate (1 wt%) and 44.25 μL of AgNO_3_ (1 M) and 9 μL of NaCl (2 M) were mixed, followed by incubating for 3 minutes in an Ultrasonic vibrator at room temperature. The solution mixture was injected into the boiling aqueous solution of L-ascorbic acid. The solution turned to bright yellow upon addition of the mixture, followed by reacting for 1 h under stirring to form uniform quasi-spherical AgNPs.

### Fabrication of dopamine-assisted Ce6-AgNPs

In brief, AgNPs were concentrated via centrifugation at 8000 rpm for 15 min, then re-dispersed in 100 mL water. 1 mL of Ce6 dissolved in DMSO (5 mM), was introduced to AgNPs solutions under continuously stirring in the dark. After 10 min, 100 mL of 0.05 mg mL^-1^ dopamine in Tris buffer (10 mM, pH 8.5) was added and the reaction solution was stirred vigorously for another 1 h. Through a rapid polymerization process, the stable dopamine-assisted Ce6-AgNPs were obtained. Then the resultant solution was purified by centrifugation at 7500 rpm for 10 min and was rinsed with water thoroughly for further use.

### Fabrication of GP-Ce6-AgNPs

Before the fabrication of GP-Ce6-AgNPs, GP-PEG-SH was prepared according to the conceptual similar procedure. In brief, the aldehyde groups of GP reacted with amino groups of amino-terminated thiolated polyethylene glycol (HS-PEG-NH_2_, MW 2000) to form a Schiff base which was further reduced by NaBH_4_ to form a stable structure (HS-PEG-GP) 45. Subsequently, the stable HS-PEG-GP could be easily conjugated with the exposed dopamine quinone on the PDA outer surface via Michael addition reaction 46, 47. Firstly, 150 mg of GP, together with 50 mg of H_2_N-PEG-SH were dissolved in 5 mL DMF and then reacted for 6 h at 70 °C under continuously stirring, followed by 1.42 mg of NaBH_4_ added for another 12 h at room temperature to obtain the stable GP-PEG-SH. Finally, 1 mL of the prepared GP-PEG-SH was introduced to the Ce6-AgNPs which was re-dispersed in 50 mL Tris buffers (10 mM, pH 8.5) in advance. The dispersion was continuously stirred at room temperature overnight to obtain the stable GP-tagged Ce6-AgNPs (GP-Ce6-AgNPs). Subsequently, the resultant solution was purified by centrifugation at 7000 rpm for 10 min to remove residual reactants. Then GP-Ce6-AgNPs were re-dispersed in deionized water and stored at 4 °C in the dark for further use.

### Characterization of GP-Ce6-AgNPs

To verify the successful fabrication of GP-Ce6-AgNPs, important components (Ce6, PDA, GP) were sequentially characterized. The morphology of GP-Ce6-AgNPs was observed using TEM. The particle size and ζ potential of AgNPs, Ce6-AgNPs, GP- Ce6-AgNPs were detected by DLS. The phenol-sulfuric acid method was performed to verify the orientation of the tagged GP molecules. Briefly, phenol was dissolved in deionized water and made up into 80% of solutions. To 200 μL of nanoagents in an Eppendorf (EP) tube was added 500 μL of sulfuric acid (98%). Then 100 μL of 6% phenol freshly prepared was introduced to the mixture. After vortex for 1 min, the mixture was heated for 15 min in a boiling water bath. 200 uL of the resultant solution was carefully transferred into a 96-well microplate and spectra of 300-800 nm were recorded using the microplate reader. It is worth mentioning that the absorption peak at 645 nm, which also confirms the successful conjugation of Ce6 molecules. The amounts of linked GP and loaded Ce6 can be quantified based on the corresponding calibration absorption curves.

GP and Ce6 loading calculations: The GP-Ce6-AgNPs concentration was calculated from the amount of AgNPs in terms of no centrifugal loss during the experimental procedure. Firstly, the radius (r) of each AgNPs was obtained by analyzing TEM pictures, ca. 22.5 nm. Subsequently, the weight of each AgNPs was calculated using the equation: m_AgNPs_ = *ρ*_Ag_·(^4^/_3_·πr^3^), ca. 5.0 × 10^-16^ g. The total weight of silver was determined as: m_total_ = M_Ag_·(CV)_AgNO3_, ca. 4.8 × 10^-3^ g. Thus, the theoretical maximum number of AgNPs could be estimated from the ratio of m_total_ and m_AgNPs_, ca. 9.5 × 10^12^ particles. Theoretically speaking, the final GP-Ce6-AgNPs concentration in 5 mL samples was calculated as 1.9 × 10^12^ particles/mL.

The loading number of GP or Ce6 molecules attached to the GP-Ce6-AgNPs was obtained respectively from the corresponding calibration absorption curves. Briefly, the linked GP concentration was estimated as ca. 90.9 μg/mL from Fig. S2B and the actual number of GP molecules per milliliter was determined as: N_GP_ = N_A_·(m/M)_GP_, ca. 5.5 × 10^16^ molecules/mL (N_A_ = 6.02 × 10^23^ mol^-1^). Thus, the amounts of loaded GP were calculated from the ratio between the concentrations of linked GP and the GP-Ce6-AgNPs concentrations, ca. 2.9 × 10^4^ molecules/particle. Similarly, the loaded Ce6 was estimated as 180.5 μM from **Figure S2D**. The actual number of GP molecules per milliliter and final amounts of loaded GP attached to GP-Ce6-AgNPs was calculated as ca. 1.1 × 10^17^ molecules/mL and 5.7 × 10^4^ molecules/particle.

### ROS generation ability of GP-Ce6-AgNPs

The ROS generation performance was evaluated by using commercial singlet oxygen (^1^O_2_) probe 9,10-Anthracenediyl-bis(methylene)-dimalonic acid (ABDA). The generated ^1^O_2_ can be harvested by ABDA, leading to its fluorescence intensity reduction at 408 nm. Typically, ABDA was dissolved in DMSO and made up to 20 μM. A final concentration of 1 μM ABDA was added into nanoagents (30 μg/mL) suspension, then irradiated by a 655 nm laser (300 mW/cm^2^) at each 5 min interval. Besides, irradiation cycles (light for 5min and then dark for 5 min) were carried to evaluate the ability of laser-activated released^ 1^O_2_. The fluorescence intensity of ABDA was measured by fluorescence spectra at 380 nm excitation and 390-500 nm emission.

### Silver ion (Ag^+^) release under laser irradiation

The nanoagents suspensions were diluted with deionized water to the desired concentrations, then irradiated by a 655 nm laser (300 mW/cm^2^). In the subsequent three irradiation cycles (light for 5 min and then dark for 5 min), the resultant suspension was centrifuged at 13000 rpm for 30 min to ensure that nanoagents were centrifuged completely to the bottom of the EP tube. Then the obtained supernatant was detected by ICP-OES for estimating the amount of the etched Ag^+^.

### Bacterial culture

MRSA, EC and CRPA were cultured in LB medium at 37 °C in a shaking incubator (180 rpm) and harvested at 5000 rpm for 2 min. After rinsing with PBS buffer, the bacteria were re-suspended in PBS for further use. The concentration of bacteria was monitored by measuring the optical density at 600 nm (OD_600_).

### Preparation and characterization of GP-Ce6/MB-AgNPs

To investigate the targeting ability of GP-Ce6-AgNPs to bacteria, a classic Raman reporter, 4-Mercaptobenzonitrile (4-MB) was introduced into the surface of AgNPs. Briefly, 1 mL of Ce6 dissolved in DMSO (5 mM), followed by 200 μL of 4-MB dissolved in DMSO (10 mM) was introduced to re-dispersed AgNP solutions (100 mL) under continuously stirring in the dark. After 10 min, 100 mL of 0.05 mg mL^-1^ dopamine in Tris buffer (10 mM, pH 8.5) was added and the reaction solution was stirred vigorously for another1 h to obtain 4-MB co-loaded Ce6/MB-AgNPs. A similar process was performed to obtain a GP-Ce6/MB-AgNPs. Raman spectra of 4-MB, Ce6, GP, Ag@PDA, and GP-Ce6/MB-AgNPs were recorded on a confocal Raman microscope (Renishaw) with a 532 nm laser excitation (30 mW), 50× objective lens, and an exposure time of 1 s.

### *In vitro* SERS imaging of bacteria

For the SERS imaging of bacteria, the bacteria suspensions were mixed with GP-Ce6/MB-AgNPs, followed by incubation for 1 h in a shaking incubator (80 rpm) at 37 °C. After rinsing with saline three times, the suspensions were transferred onto the microscope slide and then imaged by a confocal Raman microscope with a 532 nm laser excitation (30 mW), 50× objective lens, and an exposure time of 1 s. The signals from 2200 to 2260 cm^-1^ in the Raman-silent region were analyzed through WiRE 4.2 software to observe the interaction between GP-Ce6/MB-AgNPs and bacteria.

For the SERS imaging of mixture samples of bacteria and mammalian cells, 3T3 and HeLa cells were respectively cultured in Dulbecco's modified Eagle's medium (DMEM) for 24 h (37 °C, 5% CO_2_) and used for the following experiment. Typically, the cells were rinsed with PBS and then mixed with re-suspended bacterial suspension. Subsequently, GP-Ce6/MB-AgNPs were introduced to the mixture and incubated for 1 h (37 °C, 5% CO_2_) to investigate selective interactions between cells and bacteria. Concerning bacteria and cells imaging in the same field of vision, bacteria, and cells were respectively rinsed to remove non-specifically adsorbed GP-Ce6/MB-AgNPs. Alternatively, the harvested cells were fixed in 4% formaldehyde for 30 min and re-suspended in saline for further observation. After respective treatment, the obtained bacteria and cells were placed on the microscope slide. Raman imaging was performed following the aforementioned procedures.

### *In vivo* SERS imaging of bacteria

Afterward, the targeting ability of GP-Ce6/MB-AgNPs to bacterial infection was further investigated. The infected wounds (right leg) were incubated with 100 μL of GP-Ce6/MB-AgNPs for 1 h while the left leg was treated with PBS buffer. Raman spectrum of the infected mice was collected by a confocal Raman microscope. Subsequently, Raman data at 0.5 h, 4 h, 8 h, 12 h, 24 h, 48 h were collected to explore the retention of GP-Ce6/MB-AgNPs on the surface of the wound by a confocal Raman microscope with a 532 nm laser excitation (30 mW), 5× objective lens, 50×50 steps, and an exposure time of 2 s. The signals at around 2223 cm^-1^ in the Raman-silent region were analyzed through WiRE 4.2 software.

Moreover, the mice were sacrificed and the infected tissues were treated with collagenase (type III) at 37 °C in a shaking incubator (180 rpm) for at least 4 h. After incubation, the separated bacteria and cells were isolated by density gradient centrifugation procedures and measured by a confocal Raman microscope with a 532 nm laser excitation (30 mW), 50× objective lens, and an exposure time of 2 s.

### *In vitro* antibacterial assays

To evaluate the synergistic antibacterial effect, the bacterial suspensions (10^8^ CFU/mL) were randomly assigned into eight groups including PBS (control), AgNO_3_ (2 μg/mL), Ce6 (200 μM), GP-Ce6-AgNPs (30 μg/mL) and the above four with laser irradiation respectively (10 min, 300 mW/cm^2^). All groups were incubated for 1 h at 37 °C in a shaking incubator (80 rpm). After incubation and respective treatment, the bacterial suspensions were serially diluted and then 100 µL of the suspensions were spread on the LB agar plates. The amounts of bacterial colonies were counted after culturing at 37 °C for 24 h to evaluate the antibacterial effect. The bacteria viability and antibacterial rate were calculated respectively based on the generated CFU counts.

To investigate the morphology of bacteria treated with GP-Ce6-AgNPs, samples treated with GP-Ce6-AgNPs before and after 655 nm light irradiation were characterized by SEM. To prepare bacteria samples for SEM measurement, bacterial suspensions were divided into four groups: PBS (control), PBS + Light irradiation (10 min, 300 mW/cm^2^), GP-Ce6-AgNPs, GP-Ce6-AgNPs + Light irradiation. All groups were incubated for 1 h at 37 °C in a shaking incubator (80 rpm). After respective treatment, the bacteria were collected via 3000 rpm for 2min and rinsed three times with PBS buffer. The harvested bacteria were fixed in 4% paraformaldehyde (PFA) for 4 h. After rinsing with PBS, the bacteria were re-dispersed in saline and transferred onto the silicon wafers. Furthermore, silicon wafers were sputter-coated with gold before observing by SEM.

For more insights into antibacterial property, live/dead bacterial staining assay was performed. The bacterial suspensions were divided into four groups: PBS (control), PBS + Light irradiation, GP-Ce6-AgNPs, GP-Ce6-AgNPs + Light irradiation. After incubation and respective treatment, for better staining efficiency, the bacterial suspensions were rinsed with saline thoroughly. Then suspension in saline was stained with the mixture of meilungreen and PI (Live/Dead Bacterial Viability and Counting Kits), which were kept in the dark for 20 min. Afterward, the mixture was washed five times with the saline, 10 μL of the resuspensions was dropped onto a microscope slide covered with a coverslip and then observed using a confocal laser scanning microscope (CLSM) with a × 64 oil-immersion objective (meilungreen: *λ*ex = 486 nm, *λ*em = 525 nm; PI: *λ*ex = 561 nm, *λ*em = 595 nm).

DCFH-DA fluorescence method was performed to evaluate the ROS level inner bacteria after treatment. Briefly, the bacterial suspensions (10^8^ CFU/mL) were incubated with PBS, GP-Ce6-AgNPs for 1 h at 37 °C in a shaking incubator (80 rpm). After incubation and treated with and without irradiation (10 min, 300 mW/cm^2^) respectively, the bacterial suspensions were harvested and stained with 2,7-dichlorodihydrouorescein diacetate (DCFH-DA, 20 μM) in darkness for 30 min at 37 °C and then washed five times with the saline, 10 μL of the resuspensions was dropped onto a microscope slide covered with a coverslip and then observed using a confocal laser scanning microscope (CLSM) with a × 64 oil-immersion objective (*λ*ex = 486 nm, *λ*em = 525 nm). Afterward, the fluorescence intensity of DCFH-DA in different groups including bacteria, GP-Ce6-AgNPs, and bacteria treated with GP-Ce6-AgNPs was measured by fluorescence spectra at 502 nm excitation and 510-600 nm emission.

### *In vivo* antibacterial assays

Balb/c mice (6-8 weeks) were purchased from SPF (Beijing) Biotechnology Co., Ltd and all animal procedures were conducted following the requirements for the institutional ethics committee. The MRSA-infected or PA-infected mice were built on their back with an injection of 100 μL of MRSA suspension (10^8^ CFU/mL) and CRPA suspension (10^7^ CFU/mL), respectively. After infection for 24 h, the bacteria-infected mice were divided into five groups (n = 3) including the group of PBS (control), GP-Ce6-AgNPs, PBS + light irradiation, AgNO_3_ + light irradiation, Ce6 + light irradiation, GP-Ce6-AgNPs + light irradiation. The mice were treated with 100 μL of PBS (control), AgNO_3_ (2 μg/mL), Ce6 (200 μM), GP-Ce6-AgNPs (30 μg/mL) at 24 h and 48 h post-injection and irradiated constantly for 10 min under a 655 nm laser irradiation (300 mW/cm^2^). The wound area was photographed every day and then processed by Image J software.

On the last day of treatment, the blood samples were harvested by eyeball extirpating. After centrifugation at 3000 rpm for 5 min, the serum was obtained and stored at -20 °C for further use. Subsequently, the mice were sacrificed and the harvested wound tissues were soaked in sterile saline (1 mL) to extract bacteria. The bacteria-containing solutions were serially diluted and cultured on the agar plates at 37 °C for 24 h. The bacterial colonies were counted to estimate the antibacterial effect.

Moreover, the infected tissues were fixed in 4% formaldehyde for the following H&E staining. Meanwhile, ELISA kits of TNF-α and TGF-β1 were employed to detect serum, which was processed following the protocols of the manufacturer. VEGF levels in skin tissues of the wound regions were evaluated by immunofluorescence assay. In brief, the paraffin sections were deparaffinized, rehydrated, retrieved with antigen, and subsequently blocked with 3% BSA. Then, the sections were incubated with the anti-VEGF antibody at 4 °C for 24 h, followed by incubation with the Cy3 labeled secondary antibody in the dark for 50 min. Finally, the sections were stained with DAPI to locate the nucleus; after quenching the autofluorescence of the tissue, fluorescence images were observed using a fluorescence microscope (DAPI: *λ*ex = 330-380 nm, *λ*em = 420 nm; Cy3: *λ*ex = 510-380 nm, *λ*em = 590 nm).

### Biological safety evaluation

For toxicity assessment *in vitro*, the 3T3 and HeLa cells were employed in a colorimetric MTT assay. Typically, the cells were respectively cultured in 96-well plate for 24 h (37 °C, 5% CO_2_) and followed by incubation with 20 μL of GP-Ce6-AgNPs (0, 1.25, 2.5, 5, 10, 20, 30, 40 μg/mL) for another 20 h. Subsequently, 10 μL of MTT (5 mg/mL) was introduced to the well and incubated for 4 h (37°C, 5% CO_2_). Then, 120 μL of DMSO was added to lyse the cells. Finally, the optical density at 490 nm (OD_490_) was measured by the microplate reader to estimate the cell viability. To evaluate the biocompatibility *in vivo*, 100 of GP-Ce6-AgNPs (30 μg/mL) were injected into the subcutaneous tissue of healthy mice and treated with or without a 655 nm laser irradiation (10 min, 300 mW/cm^2^). Similar experiments performed with PBS buffer were regarded as the control group. After the respective treatment for 14 days, the injected area was photographed. The tissue at the injection site and major organs were harvested for H&E staining.

### Statistical analysis

All experimental data were presented as the mean ± standard deviation of three or five independent tests. The images were performed by Image J software. The statistical analyses were processed by Origin software and Statistic Package for Social Science (SPSS). p < 0.05 (*), p < 0.01 (**), and p < 0.001 (***) were conducted to indicate statistical difference.

## Supplementary Material

Supplementary figures and tables.
