# Supplementary material for: Photoactive Silver Nanoagents for Backgroundless Monitoring and Precision Killing of Multidrug-Resistant Bacteria
Source: Nanotheranostics. 2021 Jun 1;5(4):472–87. doi: 10.7150/ntno.62364 (PMC8210445; doi:10.7150/ntno.62364)
Supplement: Supplementary file 1 — Supplementary figures and tables. [file ntnov05p0472s1.pdf]

Electronic Supplementary Material

Photoactive Silver Nanoagents for Backgroundless Monitoring and Precision Killing of Multidrug-Resistant Bacteria

Zhiwen Xu[1], Cai Zhang[1], Yunjian Yu[3+], Wenshuai Li[1], Zhuang Ma[3], Jingjing Wang[2], Xinge Zhang[3], Hongmei Gao[2✉], and Dingbin Liu[1✉]

1. State Key Laboratory of Medicinal Chemical Biology, Research Center for Analytical Sciences, Tianjin Key Laboratory of Biosensing and Molecular Recognition, College of Chemistry, Nankai University, Tianjin 300071, China.
2. Department of Intensive Care Unit, Key Laboratory for Critical Care Medicine of the Ministry of Health, Emergency Medicine Research Institute, Tianjin First Center Hospital, School of Medicine, Nankai University, Tianjin 300071, China
3. Key Laboratory of Functional Polymer Materials of Ministry of Education, Institute of Polymer Chemistry, College of Chemistry, Nankai University, Tianjin 300071, China

† Present address: Key Laboratory of Biomedical Polymers of Ministry of Education, Department of Chemistry, Wuhan University, Wuhan 430072, China

✉ Corresponding authors: Hongmei Gao, E-mail: ghm182@163.com. Dingbin Liu, E-mail: liudb@nankai.edu.cn.

This file includes:

Experimental materials

Instrumentation

Figure S1. UV-Vis absorbance.

Figure S2. Quantitative analysis of GP and Ce6.

Figure S3. FL intensity of GP-Ce6-AgNPs under continuous irradiation times.

Figure S4. Ag^+ measurement with different laser power density.

Figure S5. SERS stability.

Figure S6. SERS images of GP-Ce6/MB-AgNPs treated MRSA and EC.

Figure S7. Bodyweight of MRSA and CRPA infected mice with different treatments.

Table S1. In vitro relative antibacterial rate.

Table S2. Relative wound area (S/S_0) of different treatment groups.

Table S3. In vivo relative antibacterial rate.

Experimental materials

Silver nitrate ($AgNO_3$), trisodium citrate (TSC), L-ascorbic acid (AA), Sodium borohydride ($NaBH_4$), dopamine hydrochloride (DA), sulfuric acid (H_2SO_4, 98%), phenol, ethanol, tris(hydroxymethyl)aminomethane (Tris), 4-Mercaptobenzonitrile (98%), amino-terminated thiolated polyethylene glycol (HS-PEG-NH_2, MW 2000), chlorin e6 (Ce6), glucose polymer (GP) of poly[4-O-(α-Dglucopyranosyl)-D-glucopyranose], collagenase (type III), 3-(4,5-dimethyl-2-thiazolyl)-2,5diphenyl-2-H-tetrazolium bromide (MTT), 9,10-anthracenediyl-bis (methylene)dimalonic acid (ABDA), 2,7-dichlorodihydrouorescein diacetate (DCFH-DA) were obtained from major suppliers such as Sigma-Aldrich (Shanghai, China) and Aladdin Biotechnology Co., Shanghai, China.

Methicillin-resistant *Staphylococcus aureus* (MRSA, ATCC 29213), *Escherichia coli* (EC, ATCC 25922), and Carbapenem-resistant *Pseudomonas aeruginosa* (CRPA) were obtained from the National Center for Veterinary Drug Safety Evaluation, College of Veterinary Medicine, China Agricultural University. The 3T3 and HeLa cells were provided by the Shanghai Institute of Life Sciences, Chinese Academy of Sciences (Shanghai, China). Dulbecco's modified eagle medium (DMEM), fetal bovine serum (FBS), penicillin/streptomycin, and trypsin were all purchased from GIBCO (Grand Island, NY, U.S.A.).

Instrumentation

The morphology of nanoagents was measured by transmission electron microscopy (TEM; Hitachi, HT7700). The Zeta potential and dynamic light scattering (DLS) measurements were performed with a Malvern Zetasizer (Nano ZS). The spectra were collected respectively by UV-Vis spectrophotometer (Hitachi, U-3900) and Fluorescence spectrophotometer (Hitachi, F-4600). The amount of silver ions was recorded by inductively coupled plasma-optical emission spectrometry (ICP-OES, SpectroBlue). Raman spectra and SERS images were recorded on a confocal Raman microscope (Renishaw) of 532 nm (He-Ne laser) laser under a 50× objective lens. Scanning electron microscopy (SEM) images were obtained by electron microscope (Shimadzu, SS-550) with a voltage of 15 kV. Fluorescence images were acquired using a laser confocal fluorescence microscope (Nikon).

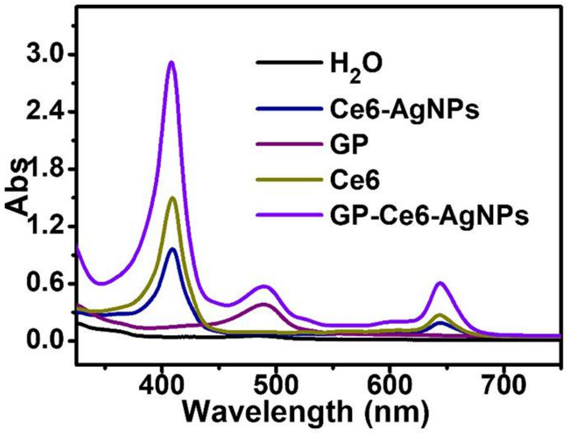

Figure S1. UV-Vis absorbance. UV-Vis absorbance of H_2O, Ce6, GP, Ce6-AgNPs and GP-Ce6-AgNPs treated with phenol-sulfuric acid.

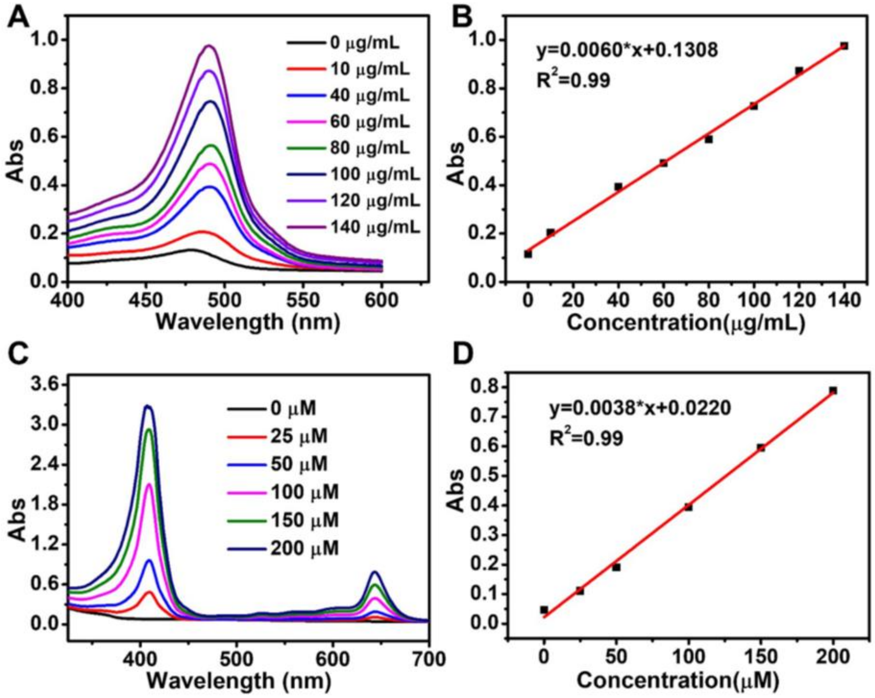

Figure S2. Quantitative analysis of GP and Ce6. (a) UV-vis absorption spectra of GP (concentration: 0, 10, 40, 60, 80, 100, 120, 140 μg/mL) treated by phenol-sulfuric acid and (b) corresponding calibration curve processed from absorption peak at around 490 nm. (c) UV-vis absorption spectra of Ce6 (concentration: 0, 25, 50, 100, 150, 200 μM) treated by the same amount of phenol-sulfuric acid and (d) corresponding calibration curve processed from absorption peak at around 645 nm.

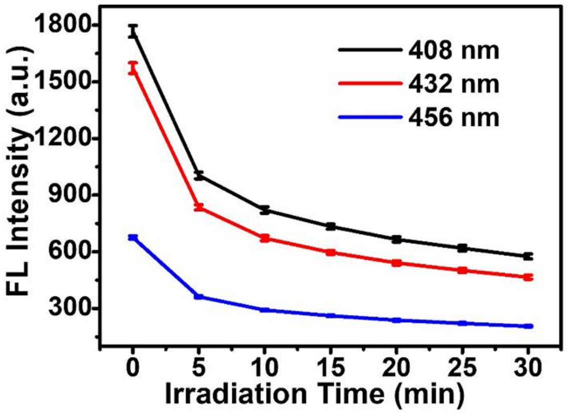

Figure S3. FL intensity of GP-Ce6-AgNPs under continuous irradiation times. FL intensity of GP-Ce6-AgNPs treated ABDA after exposing to continuous irradiation times at peak of 408, 432, and 456 nm.

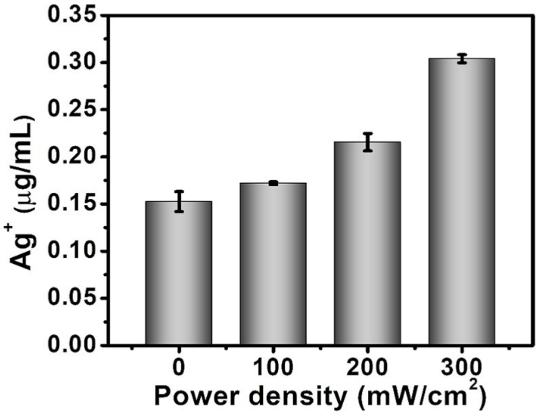

Figure S4. Ag^+ measurement with different laser power density. The amount of Ag^+ measured by ICP-OES with different laser power density for 10 min.

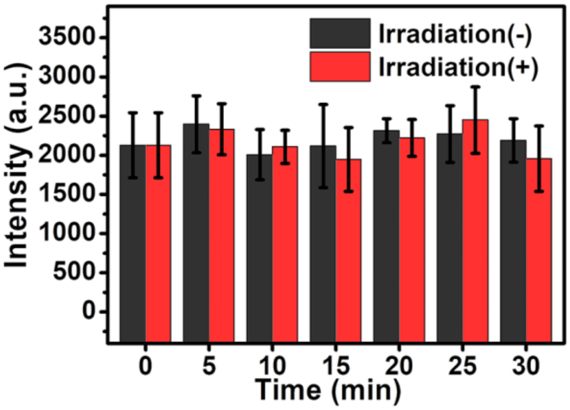

Figure S5. SERS stability. SERS intensity at round 2223 cm^{-1} of GP-Ce6/MB-AgNPs treated without and with a 655 nm laser irradiation (10 min, 300 mW/cm^2) over time.

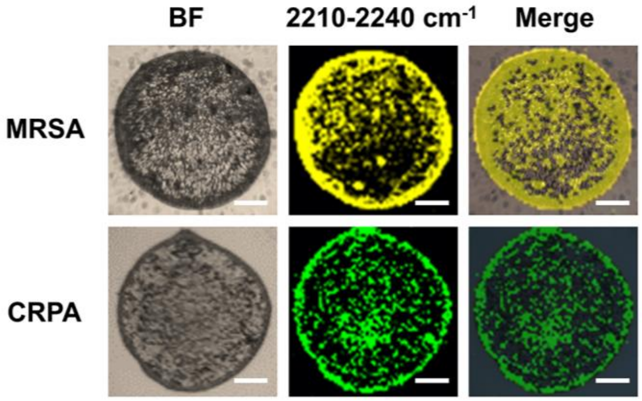

Figure S6. SERS images of GP-Ce6/MB-AgNPs treated MRSA and EC. SERS mapping images of GP-Ce6/MB-AgNPs containing MRSA, EC by using a confocal Raman microscope (Renishaw) of 532 nm (He-Ne laser) laser (30 mW, 2 s). Scale bar: 200 μm. SERS images were processed from Raman silent range of 2210-2240 cm^{-1} by WiRE 4.2 software. Pseudo-colors yellow and green respectively represented the signals of MRSA and EC.

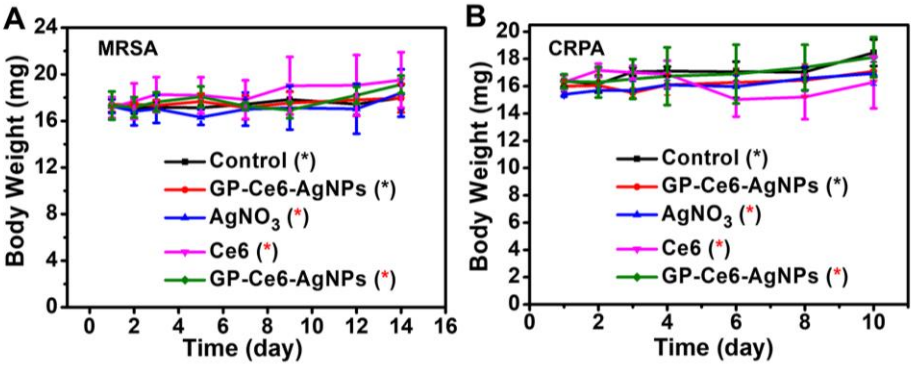

Figure S7. Bodyweight of MRSA and CRPA infected mice with different treatments. The red * represented groups treated with 655 nm irradiation (10 min, 300 mW/cm^2) while the black * represented no irradiation treatment.

Table S1. In vitro relative antibacterial rate. In vitro relative antibacterial rate of different treatment groups. The PBS treated bacteria (represented as "--" in the table) without irradiation was termed as control.

	**Control**		**$AgNO_3$**		**Ce6**		**GP-Ce6-AgNPs**	
**Irradiation**	**(-)**	**(+)**	**(-)**	**(+)**	**(-)**	**(+)**	**(-)**	**(+)**
**MRSA**	--	10.3%	27.2%	26.0%	24.3%	78.8%	17.2%	99.6%
**EC**	--	5.5%	31.8%	32.5%	18.8%	70.1%	14.3%	98.8%

Table S2. Relative wound area (S/S_0) of different treatment groups. The PBS treated bacteria without irradiation was termed as control. The red * represented groups treated with 655 nm irradiation (10 min, 300 mW/cm^2) while the black * represented no irradiation treatment.

	**Control (*)**	**GP-Ce6-AgNPs (*)**	**$AgNO_3$ (*)**	**Ce6 (*)**	**GP-Ce6-AgNPs (*)**
**MRSA**	59%	56%	36%	31%	14%
**CRPA**	67%	65%	49%	42%	18%

Table S3. In vivo relative antibacterial rate. In vivo relative antibacterial rate of different treatment groups. The PBS treated bacteria (represented as "--" in the table) without irradiation was termed as control. The red * represented groups treated with 655 nm irradiation (10 min, 300 mW/cm^2) while the black * represented no irradiation treatment.

	**Control (*)**	**GP-Ce6-AgNPs (*)**	**$AgNO_3$ (*)**	**Ce6 (*)**	**GP-Ce6-AgNPs (*)**
**MRSA**	--	8.1%	67.0%	65.5%	96.8%
**CRPA**	--	14.0%	62.7%	87.7%	93.6%
